# The relationship between young football players’ psychological health resources and the psychological quality of their football experiences: A cross-sectional study

**DOI:** 10.1371/journal.pone.0305978

**Published:** 2024-08-23

**Authors:** Yngvar Ommundsen, Andreas Ivarsson, Bente Wold, Siv Gjesdal, Bård Erlend Solstad

**Affiliations:** 1 Department of Sport and Social Sciences, Norwegian School of Sport Sciences, Oslo, Norway; 2 Halmstad University, Centre of Research on Welfare, Health and Sport, Halmstad, Sweden; 3 Department of Health Promotion and Development, University of Bergen, Bergen, Norway; 4 Department of Sport Science and Physical Education, University of Agder, Kristiansand, Norway; University of Study of Bari Aldo Moro, ITALY

## Abstract

Studies taking a person-centred statistical approach when examining young peoples` psychological experiences in sport is scarce. The main aim of the present study was to examine the relationships between young football players’ psychological health resources and the psychological quality of their football-specific experiences. Data for this cross-sectional study was collected as part of the [BLINDED] arm of the larger Promoting Adolescence Physical Activity (PAPA) multi-centre project [1]. The sample consisted of young [BLINDED] male (*n* = 814), female (*n* = 576), grassroots football players between the ages of 10 and 15 years (*M* = 12.5 years, *SD* = 1.1 years). We performed a latent profile analysis using M*plus* 8.4 using a robust maximum likelihood estimator (MLR). Players with the most resourceful psychological health profile experienced more coach social support (mean = 4.38) than did those with a less well-off resourceful profile (mean = 3.79) and those with the least well-off profile (mean = 3.28). Players with the most resourceful profile also felt a stronger sense of unity among their teammates and they enjoyed football more than those least well off (mean = 4.43 vrs. mean = 3.12 and mean = 4.74 vrs 3.50. respectively). Parallel between-profile differences were also found for the players’ general health resources including perceived life satisfaction, general health and family affluence as covariates. Findings suggest that variations in young players’ psychological health profiles and their general health resources play a role in the quality of their football-specific psychological experiences.

## Introduction

An abundance of studies has focused on potential psychological benefits and risks of taking part in organised sport. [2–5]. However, while research has pointed to the role of family capital as an enabling resource for children’s sports participation [6, 7], there are a lack of studies in which differential psychosocial health resources characterising young participants are modelled as a premise for the level of quality of their sport-specific experiences. [8–10]. Rather, studies have typically applied “a variable-centred approach, assuming the relationships between predictors and outcomes are homogenous across a population” [9].

A person-centred approach, in contrast, is driven by the assumption that the population is heterogeneous with respect to the relationships between psychosocial health attributes and the quality of their sport-specific experiences [9]. The latter approach invites a categorization of individuals into differential profiles, which can be modelled to represent a set of psychological health attributes that impact on the quality of the young players’ football-specific experiences.

Taking a person-centred approach, the purpose of the current study is to explore whether differential psychosocial health resources characterising young participants systematically relate to the quality of their sport-specific psychological experiences when taking part in organised youth football. [9, 11, 12].

Previous research has focused on different psychosocial outcomes of organised sport participation. Positive exemplars include high self-esteem, positive emotional states, and subjective vitality. [2, 3, 13]. Rather than considered outcomes, these may better be modelled as generalized psychological pre-conditions contextualized to the players’ daily life holding potential *positive* implications for the quality of their sport-specific participation experiences [9].

Conversely, negative emotional states, low general self-esteem, a reduced sense of subjective vitality. [2, 14–17] as well as psychological difficulties [5, 18] may be modelled to represent psychological pre-conditions with potential *negative* implications for the quality of their participation [9] as well as sport continuation [18].

Indeed, [5] observed that after adjusting for psychological difficulties at age 8, children who dropped out of sport between the ages of 8 and 10 years had more total difficulties and more internalising problems at 10 years of age, as opposed to children who maintained regular participation in sport. Children who engage in prolonged participation in sports may possibly possess a greater range of psychosocial self-regulation skills at the onset, which serve to generate more positive sport-specific experiences and protect them against social and emotional difficulties [5].

There is evidence that psychosocial as well as educational forms of capital within the family influence caretakers’ ability to provide surplus psychological energy and well-being to their offspring through increased capacity for pro-social communication and monitoring of self-regulation skills, as well as for providing social support [19, 20].

Young people being cared for, properly monitored, socially regulated, and supported by parents during early childhood typically develop cognitive and behavioural self-regulation capacities including ability to control behaviours, emotional reactions, and social interactions [19, 21]. This, in turn, would be expected to fuel the offspring’ own psychological health resources, such as self-esteem, subjective vitality, and positive affective states [22, 23].

Young people characterized by surplus psychological health resources may also be well prepared to meet with psychosocial expectancies within the sport environment in terms of self-regulatory development, constructive emotional reactions and social interactions, as well as with respect to following team rules and regulations [21]. Consequently, they would be expected to experience their sport participation positively as indicated by a stronger sense of enjoyment, as well as having a sense of being socially united and supported [6, 24], which in turn may increase the likelihood of thriving and remaining in [25].

In contrast, families with less psychosocial capital or resources may be at risk for developing parental distress and experiencing lack of psychological energy among family caretakers resulting in diminished capacity for prosocial communication, monitoring of their off-spring’s self-regulation skills, and provision of social support [19, 20].

Consequently, a coercive or dismissive communication style, lowered parental monitoring, and reduced psychological care for their offspring may take effect which may result in a reduced cognitive and behavioural self-regulation capacities and lower level of psychological energy for their offspring [19, 26]. Psychosocial circumstances like these are likely to elicit negative implications for their psychological health resources. An increase in antisocial behaviours generated by reduced impulse control, lower level of self-esteem, vitality, and reduced positive affective states and social withdrawal may take effect [6, 18, 21, 23].

Reduced self-regulatory capacities may, in turn, be expected to run counter to psychological expectancies from the sport environment when it comes to the young players’ social and sport-specific functioning, including capacity to react and interact emotionally in a socially defensible manner, as well as follow team rules and regulations [21].

For these young people, a reduced participation in sport may be the result [9]. Should they continue their engagement in sport, they may come to experience reduced social support and acceptance from significant others such as coach and peers, resulting in a weaker level of social unity with teammates as well as a reduced sense of enjoyment when taking part [24, 27].

Caretakers’ differential capacity to provide surplus psychological energy and well-being to their offspring is also thought to impact the players’ general health resources [24, 28–30]. Hence, there is likely some co-existence between the young player’s evaluation of their psychological health accounts and their general health accounts; with life satisfaction in general and general health being typical exemplars.

With respect to age, there is evidence that a formally regulated child-centrism [31] within the [BLINDED] sport model and adopted by the [BLINDED] Football Federation is under pressure at the grassroots level to emphasise results over participation.

Consequently, with increasing age, participants may be met with stronger social expectations for social and sport-specific self-regulation capacity and ability to socially adapt according to rules and regulations within the sport environment both off and on the pitch [31]. Due to enhanced competitive pressure and early sport specialization [32], even young players may be met with such expectations.

Using a person-centred approach [9], we examined whether a sample of young [BLINDED] grassroots football players can be identified to represent different psychological health resource profiles encompassing general self-esteem, sense of subjective vitality, and positive and negative affect [6, 22, 33]. Second, we examined the players’ perceptions of the quality of their football-specific psychological experiences including perceived social support and unity in football and football enjoyment across the psychological health resource profiles. Third, we examined whether players` scores on psychological health resource profiles were systematically related to covariates encompassing general health resources (general life satisfaction and general health) and family affluence and age.

The following research questions were raised: 1. Can distinct profiles encompassing players` psychological health resources comprising vitality, general self-esteem, positive and negative affect be identified? 2. Do players with higher scores on the psychological health resources profiles report more positive football-specific psychological experiences than those with lower profile scores? 3. Do players’ scores on their psychological health profile covary with their general health resource scores (general life satisfaction and general health), family affluence and age?

## Materials and methods

### Participants and procedures

Data for this cross-sectional study stem from the [BLINDED] arm of the larger Promoting Adolescence Physical Activity (PAPA) multi-centre project [1]. The sample consisted of young [BLINDED] male (*n* = 814), and female (*n* = 576), grassroots football players between the ages of 10 and 15 years (*M* = 12.5 years, *SD* = 1.1 years) playing 7 or 11-aside football. Inclusion criteria were maximum two coaches per team, minimum one training session per week, and teams were playing formal games. A marginal number of 10-year old players being involved in 5-aside football with no formal games were excluded. Most of the players had been involved with their current team for an average of 4.5 seasons (*SD* = 2.2). Upon accepting the invitation, coaches forwarded an information sheet to players and their parents/legal guardians who were asked to indicate consent.

The data collection took place before or after a training session, and trained data collectors administered the questionnaires. In most cases, the players completed the questionnaire in the club house, or another facility near the training pitch.

The [BLINDED] Centre for Research Data approved the project before its commencement. The data did not include sensitive health information. Hence, their approval required only passive consent (code of approval 2010/271). Parents or legal guardians were asked to give the project leader a verbal (telephone) or written (e-mail or letter) refusal of participation if they did not want their child to participate. Participants were informed that consent could be withdrawn at any point [1], and the option of opting out was given directly to the participants themselves.

#### Measures

General self-esteem was measured by the Marsh’s Self-Description scale [34; e.g. “I have a lot to be proud of”]. The five items referred to how they felt during the last month. Responses were indicated on a 5-point Likert scale anchored by not at all agree (1) and totally agree (5). Confirmatory Factor Analysis (CFA) using Mean-Adjusted Maximum Likelihood (MLR) estimator yielded very good fit indices. (X^2^/df ratio CMIN/DF) = [df = 5, N = 1397] = 4.97, p = .41; Comparative Fit index (CFI) = 1.00; Tucker-Lewis Index (TLI) = 1.00; Root Mean Square Error of Approximation (RMSEA) = .00 [.00-.04], and Standardized Root Mean Square Residual (SRMR) = 0.001). Factor loadings were significant and ranged from .98 to .99. Cronbach alpha was .73.

Subjective vitality was measured by the subjective vitality scale [35; e.g. “I nearly always felt alert and awake”]. The stem was how they felt during the last month. Responses on the five-item scale were indicated on a 5-point Likert scale anchored by not at all agree (1) and totally agree (5). CFA analyses using MLR estimator yielded excellent fit indices. (X^2^/df ratio CMIN/DF) = [df = 5, N = 1397] = 6.28, p = .28; CFI = .99; TLI = .99; RMSEA = 0.01 [.00-.04], and SRMR = .001). All factor loadings were significant and ranged from .98 to .99. Cronbach alpha was .81.

Positive and negative affect was measured by the PANAS scale [36]. The participants were presented with a list of ten emotions and asked to indicate the extent to which they usually have these feelings; e.g. “interested” (positive affect) and “upset” (negative affect). Responses on the ten-item scale (5 item each) were indicated on a 5-point Likert scale anchored by very low extent (1) and very high extent (5). CFA analyses using MLR estimator yielded excellent fit indices for the five positive affect items: (X^2^/df ratio CMIN/DF) = [df = 5, N = 1397] = 5.58, *p* = .35; CFI = .99; TLI = .99; RMSEA = .009. [.000-.039], and SRMR = .002). All factor loadings were significant and ranged from .97 to .98. Cronbach alpha.77. CFA analyses using MLR estimator yielded excellent fit indices also for the five negative affect items: (X^2^/df ratio CMIN/DF) = [df = 2, N = 1397] = 2.59, p = .27; CFI = .99; TLI = .99; RMSEA = .001 [.000-.057], and SRMR = .001). Factor loadings were significant and ranged from .97 to .98. Cronbach alpha was.72.

Social support in football was measured using the Brief Social support scale [37; e.g. “My coach can really be counted on to care, no matter what happen”]. The stem was how they felt when playing on the team the last 3-4 weeks. Responses on the three-item scale were indicated on a 5-point Likert scale anchored by not at all agree (1) and totally agree (5). Given the three-item scale ordinary CFA estimates of the scale cannot be estimated, and all values are set to zero. Loadings ranged from .42 to .94. Cronbach alpha.70.

Social unity in football was measured by a scale compiled following a review of the sport and social psychology literature [e.g.,^38^; e.g., “We trust each other”]. The stem was how they generally felt when playing on the team. Responses on the six-item scale were indicated on a 5-point Likert scale anchored by not at all agree (1) and totally agree (5). A coach version of the scale has previously been used revealing good psychometric estimates [39]. CFA analyses using the MLR estimator yielded excellent fit indices: (X^2^/df ratio CMIN/DF) = [df = 9 N = 1397] = 15.098, p = .88; CFI = .98; TLI = .97; RMSEA = .02. [.00- .04], and SRMR = .01). Factor loadings were significant and ranged from .99 to 1.00. Cronbach alpha was.89.

Enjoyment in football was measured by the Intrinsic Motivation Inventory (IMI), using the enjoyment sub-dimension [40; e.g. “I found that time flew by when I was playing football”]. The stem was how they felt while playing football on their team during the past month. Responses were indicated on a 5-point Likert scale ranging from not at all agree (1) to totally agree (5). The CFA showed that the Enjoyment scale yielded excellent fit indices (X^2^/df ratio CMIN/DF) = [df = 2, N = 1397] = .00 p = .00; CFI = 1.00; TLI = 1.07; RMSEA = .00 [.00-.00], SRMR = .00). Factor loadings ranged from .94 to .97. Cronbach alphawas.81.

Self-rated general life satisfaction was measured using the Cantril ladder, a visual analogue 11-point scale for rating how adolescents feel about their life at present (worst possible life = 0 to best possible life = 10). This ladder is easily understood and a reliable instrument in adolescent populations [41, 42].

Self-rated general subjective health was assessed by asking adolescents: “Would you say your health is”? Very good, good, quite good, bad [30]. This one-item format has been shown to reflect young peoples’ appraisals of several aspects of their physical health and non-physical health dimensions [30].

Family affluence was measured by the perceived wealth indicator which reflects the concept of relative wealth and is measured using the following question: ‘How well off do you think your family is? [43]. The responses were given on a 5-point Likert scale, ranging from 1 (particularly well of financially) to 5 (not well of financially). The scale has previously shown evidence of good sensitivity [44] and good consistency between children’s and parents’ self-reports have been confirmed [45].

Age was measured through self-report of date, month, and year of birth.

#### Statistical analysis

Descriptive statistics and bivariate correlations are shown in Table 1. The fit of the measurement models for each latent construct in the model was estimated followed by latent profile analysis (LPA). For all profile variables and sport-specific psychological outcome variables. computed mean scores were used r to represent the respective constructs in the main analyses.

**Table 1:** Summary of intercorrelations (Pearson), scale ranges, means and standard deviations.

	Mean	SD	Scale range	1	2	3	4	5	6	7	8	9	10.
1. Self-esteem	4.01	.63	1-5	1	.54**	.55**	-.45**	.37**	.35**	.38**	.48**	.26**	.18*
2. Vitality	3.93	.68	1-5		1	.61**	-.34**	.35**	.45**	.32**	.51**	.29**	.18**
3. Positive affect	4.19	.59	1-5			1	-.33**	.37**	.44**	.40**	.43**	.26**	.17**
4. Negative affect	1.97	.65	1-5				1	-.25**	-.22**	-.22**	-.39**	-.15**	-.12**
5. Social support	3.99	.73	1-5					1	.45**	.28**	.22*	.10**	.06*
6. Social unity	4.01	.70	1-5						1	.32**	.29**	.17**	.10**
7. Enjoyment	4.69	.43	1-5							1	.25**	.16**	.15**
8. Life satisfaction	8.10	1.62	1-10								1	.31**	.23**
9. Health	3.37	.72	1-5									1	.18**
10. Fam affluence	-	-	1-5										1

* Correlation is significant at 0.05 level (two-tailed)

** Correlation is significant at 0.01 level (two-tailed)

We performed a (LPA) using a robust maximum likelihood estimator (MLR) in M*plus* 8.4 [46]. In the LPA framework, posterior profile probabilities are estimated to define each participant’s class belonging (i.e., participants are included within the profile where they have the highest probability to belong [47]. From a person-centred analytical approach, a LPA analysis with three profiles were estimated based on the four psychological health resources; *subjective vitality*, *general self-esteem*, and *positive and negative affect*. Within the analyses, a sequence of nested models, starting with one profile, was compared to examine if more complex models (i.e., a model containing one additional profile) showed better fit to the data than more parsimonious ones. Models with one-to-five classes were tested to identify the optimal number of profiles based on statistical criteria and substantive meaning.

We inspected several criteria to determine the best-fitting model [47]. More specifically, lower values on the Bayesian Information Criterion BIC [47] and the sample-size adjusted BIC [48] indicated a better model fit.

Further, statistically significant values on the adjusted Lo-Mendell-Rubin test (LMR [49] and the bootstrap likelihood ratio test (BLRT) indicated that the current model solution fit the data better than a model solution with one less profile specified. Lastly, we inspected the entropy criterion which indicate how accurately the participants fit their respective profiles [50]. Values closer to 1 indicate better accuracy [51]. In combination with the inspection of the statistical model fit indices, we also made an expert decision to select the most meaningful solution from a theoretical and substantive perspective. In this process, we were guided by the recommendations to ease the interpretation and presentation of the results by avoiding solutions with too many latent profiles [52].

In the second step of the LPA analysis, we performed the three-step approach to test potential differences in the specified covariates as well as outcomes between the profiles in the selected solution [53]. More specifically, we used the BCH approach to test differences between the profiles for the continuous variables and the DCAT approach for the dichotomous variables. In the current study, *p*<.05 was selected to indicate statistically significant results. Also, we calculated Cohen’s d effect sizes [54] to indicate the magnitude of the differences between profiles for the continuous variables. For the dichotomous variables risk differences were calculated.

## Results

### Descriptive analyses

Table 1 reveals that the players generally reported high levels of general self-esteem, vitality, and positive affect, as well as low levels of negative affect. They also reported high levels of social support, social unity, and enjoyment when engaged in their football environment. Further, the profile variables (e.g., self-esteem, vitality, and positive affect) were consistently and positively correlated with the three outcome variables (range; .14 to .44, *p*<.001), whereas negative affect was negatively correlated with the three outcomes (range; -.22 to -.25, *p*<.001).

Moreover, the two covariates life satisfaction and general health were both consistently and positively related to the three outcomes (range; .09 to -.29, *p*<.001), and the profile variables revealed a consistent correlation pattern with the two covariates life satisfaction and general health; range; .-15 (negative affect- general health) to .51 (vitality-life satisfaction), *p*<.001).

#### LPA analyses

The person-centred statistical approach identified a model comprising a 3-profile player solution, in which players were statistically profiled based on the four psychological health resources (e.g., *subjective vitality*, *general self-esteem*, and *positive and negative affect*) had the best fit to the data (see Table 2). When presenting the data for this 3-profile player solution, we have used the following labelling concerning the three different profiles: *most resourceful profile (n = 430)*, *less resourceful profile (n = 615)*, and *least resourceful profile (n = 70)*. The scores for the four psychosocial health resource variables (e.g., *subjective vitality*, *general self-esteem*, *and positive and negative affect*) across the three latent profiles are provided in Table 3.

**Table 2:** Fit indices, entropy, and model comparisons for estimated latent profile analyses models (N = 1115).

Model	BIC	SSA-BIC	Entrophy	LMR	BLRT	nC < 10/5%
**2 profile**	8099.15	8057.86	0.67	.003	<.001	0/0
**3 profile**	7668.04	7610.87	0.81	<.001	<.001	1/0
**4 profile**	7610.17	7537.12	0.85	<.001	<.001	1/1

*Note*. BIC = Bayesian Information Criterion; SSA-BIC = Sample Size Adjusted Bayesian Information Criterion; LMR = *p*-value for Adjusted Lo-Mendell-Rubin likelihood ratio test; BLRT = *p*-value for bootstrap likelihood ratio test; nC < 10/5% = number of profiles with less than 10 and 5% of the cases, respectively. In the 4-profile solution, one of the profiles contained 2 participants.

**Table 3:** Mean values for study variables for the three latent profiles.

Variable	Profile 1 (*55%*) Less res	Profile 2 (7%) Least res	Profile 3 (39%) Most res		P1 vs P2	P1 vs P3	P2 vs P3
**Profile variables**							
Vitality	3.67	2.78	4.50				
General Self-Esteem	3.85	2.81	4.53				
Positive Affect	4.00	2.98	4.65				
Negative Affect	2.15	2.76	1.74				
**Outcome variables**					Cohens d ES		
Perceived Social Support in Football	3.79^a,c^	3.28^a,b^	4.38^b,c^		0.74	-0.75	1.42
Social Unity in football	3.83^a,c^	3.12^a,b^	4.43^b,c^		1.09	-0.86	-1.86
Football Enjoyment	4.16^a,c^	3.50^a,b^	4.74^b,c^		1.02	-0.90	-2.07
**Covariates**				Risk difference (%)			
Life Satisfaction (%)							
*CAT1 (high level of LS)*	58.3	10.2	93.3		48.1	-35.0	-83.1
*CAT2 (low level of LS)*	41.7	89.8	6.7				
Subjective Health (%)							
*Category1 (good SH)*	87.7	67.7	91.5		20	-3.8	-23.8
*Category 2 (poor SH)*	12.3	32.3	8.5				
Age (%)							
*10/11*	1.5	0	3.5				
*12*	34.1	33.3	45.8				
*13*	28.0	31.8	30.5				
*14/15*	36.4	34.9	20.2				
Family affluence (%)							
*Very good*	23.7	24.1	45.8				
*Good*	57.1	41.2	43.3				
*Average*	17.3	26.2	9.9				
*Not good*	1.8	8.4	1.0				

Results from the 3-step approach identified three profiles which were used for two consecutive analyses. First, we conducted a profile analysis of the psychological health resources against players’ self-reports on the psychosocial outcomes experienced in the context of grassroots youth football. These analyses revealed statistically significant differences between the three latent resource profiles for *perceived social support* (χ^2^ (2) = 180.65, *p* < .001), (*perceived social unity among teammates* (χ^2^ (2) = 254.67, *p* < .001), and *self-reports of sport enjoyment* (χ^2^ (2) = 286.65, *p* = .003). More specifically, players with the highest probability of belonging to the most resourceful profile (P1) differed from players with higher probability of belonging to any of the two other profiles, i.e. least resourceful (P2) and less resourceful (P3) in terms of reporting higher levels of social support (Cohens d ES 0.74 vrs -0.75 and 1.42, respectively), social unity among teammates (Cohens d ES 1.09 vrs -0.86 and -1.86, respectively), and sport enjoyment (Cohens d ES 1.02 vrs -0.90 and -2.07, respectively). Throughout, players with the highest probability of belonging to the least resourceful profile reported the lowest levels of social support, social unity among teammates and sport enjoyment.

Guided by our third study purpose, we examined whether there were differences between profiles in terms of scores on general health resources (e.g., self-reports of life satisfaction and subjective health), family affluence and age. Findings showed that the most resourceful profile reported higher levels of life satisfaction (> 93%) and subjective health (> 91%). Conversely, in the least resourceful profile, findings were more divergent, showing lower levels of life satisfaction (> 89%), and additionally, lower levels of subjective health (> 32%). Further, the three resourceful profile groups also diverged in terms of family socio-economy. Whereas 33% in the least resourceful profile reported average or not good, the numbers in the less resourceful and the most resourceful groups were clearly less, 19% and 11%, respectively. The opposite pattern was revealed for those reporting very good or high family affluence.

For statistical comparisons we also examined the psychosocial outcome variables across the three psychological health resource-based profiles using χ2 values and effect sizes (Cohen’s *d*) and across the general health covariates using risk differences (see Table 3).

Regarding age, the results were less clear-cut between the different profiles. Among the 12 years old, the percentage of players belonging to the most resourceful group was somewhat higher than among the two other profile groups (46% versus 33-34%), whereas their respective group representation was equal among the 13 years old. At age 14 and 15, the percentage of players belonging to the less- and least resourceful groups were clearly higher (34-36%) as compared to those in the most resourceful group (20%).

## Discussion

The main aim of this study was three-fold. First, examine the extent to which a sample of young [BLINDED] grassroots football players can be identified to represent different psychological health resource profiles. Second, investigate whether different profiles pertaining to the young players’ psychological health resources are systematically related with the quality of their football-specific psychosocial experiences. Third, examine whether players` scores on psychological health resource profiles covary with their general life satisfaction, general health, family affluence and age.

Latent profile analysis based on the indices of psychological health resources demonstrated that a three-profile solution provided the best fit to the data. Those belonging to the most resourceful psychological health profile represented close to 40 percent of the players, the less resourceful group accounted for 55 percent, and the least resourceful 7 percent. The most resourceful profile reported a higher level of psychological health resources than the less and least resourceful groups. They also reported a stronger sense of subjective vitality, general self-esteem, and positive affect in their daily life, as well as lower negative affect. In contrast, the least resourceful profile of grassroots players reported the lowest level of vitality, self-esteem, and positive affect across their daily life.

Our findings suggest that these young grassroots football players can be statistically differentiated to represent distinct psychological health profiles and the results provide support that grassroots youth football attract players which differ in their psychosocial health resources [55–57]. Viewed in isolation, these findings are encouraging by showing that young players characterized by different psychosocial resources partake in grassroots youth football. Nevertheless, they are clearly unevenly represented. The subgroup scoring lowest on psychosocial health resources is clearly those least represented. The percentage of young people belonging to this group in the general population is unknown. Irrespectively, one explanation may be that players belonging to this profile group have already left grassroots football, or that many of them never participated in football in the first place. In support for the former, [5] observed that lower psychosocial health resources as exemplified by greater psychological difficulties are experienced by young people who drop out of sport. Alternatively, children identified by lower psychosocial health resources as exemplified by conduct problems and lack of social skills seems less likely to participate in organized activities in the first place [58].

A key contribution of this study was the observation of significantly different mean scores for perceived social support, social unity, and football enjoyment among those most resourceful group of players as compared to those less resourceful, with those least resourceful scoring significantly lower than the two former profiles. Further, the pattern of results was consistent with strong effect sizes between all three respective profiles.

Apparently, young football players characterized by surplus psychosocial health resources in their daily life, may be better prepared socially and psychologically to meet with psychosocial expectancies within the team environment in terms of their ability to control and regulate their behaviours, emotional reactions and social interactions, as well as with respect to following team rules and regulations [21]. The team sport environment consisting of coaches and parents may then be in a better position to take advantage of the players’ already existing psychological skills to the benefit for common team goals [59].

As the results also suggest, the payoff for the resourceful players seems to be a more positive experience of their football participation in terms of a stronger sense of enjoyment, as well as being socially united and supported when taking part. Indeed, wanting to be part of a team or to be with friends, and gaining social recognition have been shown to make up important psychological prerequisites for the quality of young peoples’ sport motivation [60, 61].

In contrast, young players supposedly equipped with a lower level of psychosocial health resources may show reduced ability to interact socially sufficiently with their football teammates due to a lower capacity for cognitive and behavioural self-regulation to control their behaviours vis a vis teammates [6, 23]. Consequently, their ability to react and interact emotionally and socially defensible manner, as well as follow team rules and regulations may suffer [21]. The sport environment may be particularly prone to generate competitive feelings in young players, possibly eliciting aggressive feeling states among some [62]. For children with a lower capacity for cognitive and behavioural self-regulation to control their behaviours vis a vis teammates and the opposing team, such feeling states may be particularly troublesome by potentially leading to even aggressive behaviours with negative reactions and bullying from teammates and opposing team members as an end result. Negative payoffs in terms of reduced enjoyment, social marginalization, and lack of social support when taking part may come as no surprise. Indeed, competitive youth football in [BLINDED] is mainly run by volunteer coaches and parents. They may not have the capacity, possibility nor motivation to provide guidance and assistance to adjust socially players lacking in social and emotional skills and capacity for self-regulation [22, 57, 63]. Even systematically developed physical activity programs for children with social, emotional, and behavioural disabilities have shown limited effectiveness in terms of enhancing social and psychological adjustment [64].

As indicated by our findings, the net result for players equipped with a lower level of psychosocial health resources may be a lower level of enjoyment, a reduced group feeling, and lower sense of being socially supported by their significant others within the football environment. Indeed, poor sportsmanship, lack of prosocial behaviours, as well as episodes of explicit antisocial behaviour have been associated with less positive experiences in sport [65] and dropout [18]. As such, the results concerning differential perceptions of social unity, support, and enjoyment in football among players with psychological health resources may indicate a *psychological divide* to exist in this sample of grassroots youth football players.

A third purpose was to explore whether the players’ general health resources, family affluence, and age acted as covariates. We observed that the players’ general health resources comprising self-reports of life satisfaction and subjective health were also shown to differ between the profiles. Results paralleled to a great extent their profile scores on psychosocial health resources. Specifically, high life satisfaction and good subjective health were considerably more frequently reported among those in the most resourceful psychosocial health resource profile group as compared to players in the least resourceful group (risk difference P3 vs P2 = -.83.1 and -23.8, respectively). Oppositely, clearly less frequently reported among those in the least resourceful profile than among players belonging to the less resourceful profile (risk difference P1 vs P2 = 48.1 and 20.0, respectively).

In a previous study based on the same sample as the current one, [44] using a variable-centred approach found that the players rated their life satisfaction and subjective health more favourably than a nationally representative reference sample. Indeed, our analyses taking a person-centred approach seem able to provide a more nuanced picture by considering also the combination of scores on the different psychosocial health resource indicators. Apparently, using a typical variable-centred approach one may not be in the position to identify profile-specific differences, which is likely to increase the possibility of providing inflated comparison of results.

The self-reports of family affluence across the profiles revealed a parallel tendency with those provided by the psychological and general health resource scores (see Table 3). *Very good* or *good economy* was reported among 89% in the most resourceful profile group as compared to approximately 80% in the less resourceful group, and only among 65% of those belonging to the least resourceful profile. Moreover, the correlations of family affluence to the psychological and general health resource group profile variables (See Table 1, right column), revealed a consistent pattern of results in line with expectations. Family affluence is positively, albeit not strongly, correlated with all scores on the psychological and general health resource group profile variables and negative affect being negatively correlated.

Taken together, the overall pattern of results attests to the co-existence of a social divide and a psychological divide for these players in grassroots youth football. Indeed, previous studies have shown that young players from higher socio-economic backgrounds are clearly overrepresented in organized youth sport, whereas young players from families less well of socio-economically are underrepresented [7, 24]. Recent [BLINDED] research data even show that the social divide is increasing. Children and youth from homes with the highest socio-economic status now have almost twice as high a chance of participating in organised leisure activities, including sports, compared to those who come from homes with the lowest socio-economic status [66].

The findings for age were less consistent and not readily explainable. Apparently, despite having a strong sense of social support and thriving when playing football, players aged 12 to 15 years seem more disposed to leave the football context than those reporting their football experiences as less socially and emotionally satisfying. Moreover, these players also represent the most resourceful group in terms of family affluence. While high family affluence, a strong parental educational background, and family sport culture have been shown to help keep up young peoples’ affinity for organized sport participation during adolescence [66, 67], the same holds for young people’s motivation for high priority for school and good grades [68]. What is important for adolescents, if they experience a potential conflict between school and sport participation, would seem important to identify in future research.

### Strengths and limitations

A person-centred analytical approach allows for a more fine-tuned and profile-based categorization of the players. It provides the possibility to discover rather subtle differences between profile groups that would otherwise have gone unnoticed [9]. Indeed, subtle differences were observed, as indicated by the profile-specific differences between the profiles based on psychosocial health resources and the outcomes, comprising the quality of their sport-specific experiences. Moreover, by modelling variations in young football players’ psychosocial resources as potential pre-conditions for the level of quality of their football-specific psychological experiences, psychological factors usually considered outcomes of sport participation, may instead be treated as pre-requisites for such psychological outcomes. This may help further our understanding of which sub-groups of young people are positioned to benefit the most as well as less and least from taking part in organised sport, as well as for entering organized sport in the first place.

We acknowledge the cross-sectional design as a clear study limitation. Future studies would do well by combining a person-centred analytical approach with a longitudinal design. This would allow researchers to trace more in-depth over time young people coming from families characterized by different social, psychological, and economic resources, and monitor the development and stability of their psychological characteristics and health resources. To follow their different behavioural sport-specific pathways across time, including age when entering into organized sport (if entering at all), the age period of their participation as well as age leaving organised sport, coupled with the quality of their sport-specific experiences across the age range of involvement would seem pertinent to identify.

#### Take home messages

Administrators, coaches, and parents involved in team sport would do well to create psychosocial environments in which prosocial behaviour (e.g., verbally encouraging a teammate) and self-regulation skills are encouraged and reinforced, and in which all young players are socially supported and included. To this end providing an autonomy-supportive climate and supporting basic psychological needs show promise [69]. Indeed, established correlates of youth sport attrition are to a large extent social in nature [70, 71]. Hence, the use of social events, teambuilding, and positive messaging, as well as close supervision of practices and games may also prove effective in helping to enhance group cohesion, inclusion, and enjoyment among young players [1, 65].

## Conclusions

The results generally provided a clear pattern of results revealing that young football players representing different psychosocial health resource profiles reported their football participation to be significantly different in terms of the quality of their experiences. Players with the most beneficial psychological health resource profile experienced their participation in grassroots youth football clearly and significantly more positively in terms of social support than did those less - and least well of with respect to psychological health resources. They also felt a stronger sense of unity among their teammates, and they enjoyed playing football more. This same group of players also scored higher on general health resources including life satisfaction and general health and reported to be most well of in terms of family affluence. The present study provides support for using a person-centred analytical approach [72] when studying relationships between young players’ psychological health resources and the quality of their football-specific psychosocial experiences.
